# Local Application of Probiotic Bacteria Prophylaxes against Sepsis and Death Resulting from Burn Wound Infection

**DOI:** 10.1371/journal.pone.0165294

**Published:** 2016-10-25

**Authors:** Anne Argenta, Latha Satish, Phillip Gallo, Fang Liu, Sandeep Kathju

**Affiliations:** 1 Department of Plastic Surgery, University of Pittsburgh Medical Center, Pittsburgh, PA, United States of America; 2 McGowan Institute for Regenerative Medicine, Pittsburgh, PA, United States of America; Massachusetts General Hospital, UNITED STATES

## Abstract

**Objective:**

To determine if local prophylactic application of probiotic bacteria to burn wounds will prevent death in a mouse model of burn wound sepsis.

**Background:**

Infection remains the most common complication after burn injury and can result in sepsis and death, despite the use of topical and systemic antibiotics. *Pseudomonas aeruginosa* is a frequently implicated pathogen. Local application of probiotics directly to burn wounds is an attractive novel intervention that avoids the pitfalls of standard antibiotic therapies.

**Methods:**

A burn-sepsis model was established using a sub-eschar injection of bioluminescent *P*. *aeruginosa*; infection was tracked using a charge-coupled camera. Full-thickness burn injuries were placed on the dorsums of adult mice; the injured sites were then treated with vehicle (burn wound control), probiotics (*Lactobacillus plantarum* only), pathogenic bacteria (*Pseudomonas aeruginosa* only), or probiotics plus pathogen (*Lactobacillus* plus *Pseudomonas*). Animals were monitored until death/moribundity or for one week, then sacrificed. Harvested tissues were subjected to imaging and molecular assays.

**Results:**

Control and probiotic-only animals showed no mortality (100% survival) at one week. *Pseudomonas*-only animals showed > 90% mortality within 40 hours of infection. In contrast, animals treated with probiotics plus *Pseudomonas* showed less than 10% mortality. Use of bioluminescent *Pseudomonas* bacteria demonstrated that probiotic therapy inhibited septicemic accumulation of the pathogen in remote organs. In addition, probiotic therapy successfully suppressed the infection-dependent induction of TNF-α and interleukins 6 and 10 in the liver.

**Conclusions:**

Local probiotic therapy shows great potential as a valuable adjunct in the management of complicated burn injury.

## Introduction

Nearly 500,000 patients per year receive treatment for some form of burn injury per the American Burn Association [1] and each year burn injuries in the United States result in approximately 60,000 hospitalizations, with burns ranking fourth as a cause of unintentional child injury death [2]. Burn injuries also account for at least 5% of military trauma and in general inflict enormous morbidity on the afflicted. Infection following burn injury remains the most common complication in burn wound patients of all age groups and is the most cited reason for mortality, accounting for up to 60–75% of burn related deaths [3,4]. Frank sepsis is the most common cause of death in children as well as adults [5,6], and the burn wound itself has been identified as the most likely portal of entry for the invading pathogens [7].

The majority of first and second degree burn injuries do not progress to invasive infection, but the problem of burn wound infection remains critical for those patients with large total body surface area (TBSA) burns and third degree burns. In addition to violating the physical skin barrier, burns disrupt the immunological, sensory, and metabolic functions of the skin, leaving a susceptible route for bacterial invasion [3]. Burn wounds, classically considered to be sterile immediately upon injury, become colonized and infected within one week after insult, often while the patient is under direct hospital care. Burn eschar provides a protein rich, avascular environment that encourages microbial growth, while impeding the influx of host immune cells and antimicrobial agents. It has also been recently recognized that numerous bacteria (and fungi) exist within burn wounds in the form of biofilms [8]. Biofilm bacteria are typically attached to tissue or foreign-body surfaces, and display a highly divergent behavior from their planktonic (free floating) counterparts; they are highly resistant to both conventional antibiotics and to natural host immune responses, as well as being recalcitrant to detection by standard microbiological culture [9].

One of the most frequently implicated pathogens in infection-related burn complications is *Pseudomonas aeruginosa*. Part of normal gut flora, *P*. *aeruginosa* is also prevalent in nature and has been identified on many hospital surfaces, including hydrotherapy tanks, mattresses, and heathcare workers’ hands [10,11]. While healthy hosts are able to suppress *Pseudomonas*, the immunocompromised state associated with burn injuries weakens both the innate and adaptive immune defenses against Pseudomonal growth, disposing towards pathogenic invasion. *Pseudomonas* species produce a wide array of factors- adhesins, alginate, pili, flagella, lipopolysacharride, elastase, exotoxin A, exoenzyme S, hemolysins, iron binding proteins, proteases- that mediate microbial motility, adhesion, evasion, tissue destruction, and leukocyte death [3]. Because *P*. *aeruginosa* adapts rapidly, widespread and sometimes indiscriminate use of antibiotics has led to multi-drug resistant strains [12]. Moreover, *P*. *aeruginosa* is well-known to be capable of biofilm formation, which further limits effectiveness of conventional antibiotic therapy [13].

The combination of a virulent and drug-resistant pathogen in a hospitable local milieu, in the setting of functional immune impairment, makes burn wound infection especially difficult to overcome, despite aggressive use of both topical and systemic antibiotics. We hypothesized that probiotics- that is, the use of living bacteria for the benefit of the host- may represent a superior means of counteracting and prophylaxing against burn wound infection and sepsis. Probiotics are an enticing treatment alternative to antibiotics. In addition to its popularity in the lay press and food industry, probiotic therapy has been shown to mitigate infections of middle ear, bladder, gut, and urogenital tract in animal models and patients [14]. In many cases lactic acid bacteria are the probiotic agents of choice, with the ability to both outcompete pathogens and regulate the immune response, for example through inhibition of neutrophil and macrophage apoptosis and enhancement of phagocytic activity [15,16]. Probiotic therapy against *Pseudomonas* is especially appealing, with evidence that *Lactobacillus plantarum* can interfere with Pseudomonal quorum sensing, inhibit Pseudomonal biofilms, and even reduce Pseudomonal bioburden in a burn wound model [17,18]. However, the ability of a locally administered probiotic regimen to inhibit the hypermetabolic inflammatory response elicited by burn wound infection and to actually protect against sepsis and death has never been assessed. We therefore examined the ability of *L*. *plantarum* to combat *Pseudomonas*-dependent infection in a mouse model of burn wound sepsis.

## Methods

This study was approved by the University of Pittsburgh Institutional Animal Care and Use Committee (protocol #1110766). Female C57 BL/b mice, age 7–9weeks, (Harlan Laboratories; Frederick, MD) were used for the animal models described in this study. All mice acclimated for one week prior to experiments and housed individually. All experimental manipulations were performed under a sterile hood using aseptic technique.

### A non-lethal mouse burn model

A non-lethal burn wound model was first established by applying direct thermal injury with a 1 cm diameter heated round brass stamp (Granger®, Chicago, Il). Mice were anesthetized with a mixture of ketamine (80mg/kg) and xylazine (12mg/kg) delivered via intraperitoneal (IP) injection. Hair in the left paraspinal /flank region was removed using a razor and depilatory cream (Nair®, Church and Dwight, Princeton, NJ). On the day prior to burn, stamps were wrapped in aluminum foil and heated in a Fisher convection oven (Fisher Scientific, Hanover Park, Il) overnight at 80^ο^C; this allowed them to maintain core heat during transfer from oven to mouse. Burns were created under a sterile hood by applying the stamps with direct pressure for 20 seconds to the lumbar paraspinal region of anesthetized, shaved mice. Immediately after burn, mice were resuscitated with 0.5cc of sterile normal saline delivered IP. After surgery, all animals were routinely inspected three times daily for signs of pain and distress. All animals received standing doses of Buprenorphine (0.03mg/kg) at the time of surgery and three times a day for two days postoperatively, and then as needed for any animals that did show signs of persistent distress. Animals were monitored closely for signs which signaled approaching demise as per University of Pittsburgh’s IACUC’s suggestions, including palpable hypothermia, inability to ambulate and unresponsiveness to prodding by touch. If animals exhibited these signs post-infection, death was regarded to be imminent and they were euthanized immediately by CO_2_ asphyxiation process.

All animals also received DietGel ® Recovery (ClearH_2_O, Portland, ME) post-burn, in addition to standard food and water. No wound splints were used. This technique yields non-lethal, histologically verified full-thickness wounds that heal by contraction with a small 3–4mm scar within three weeks. All mice tolerated burn injury alone without complications.

### A mouse burn-sepsis model

A burn-sepsis model was established by following the burn protocol above, then delivering a one-time sub-eschar injection of a 200 μl concentration of 1x10^7^ cfu of bioluminescent *P*. *aeruginosa* Xen41 (Caliper Life Sciences, Hopkinton, MA; preparation described below) 24 hrs after the burn injury. The intensity of Pseudomonal infection was tracked and quantified daily using IVIS camera imaging of bioluminescence (described below). Animals were monitored closely until death/moribund status, then sacrificed. Animals showing no complications were sacrificed at one week after burn wounding. Organs (liver, lung, heart), blood, and wound tissue were collected at time of sacrifice and underwent IVIS imaging and storage in RNALater (Ambion®, Austin, TX) for subsequent molecular analyses. This model consistently results in moribund state within two days after Pseudomonal injection, with consistent translocation of bioluminescent *P*. *aeruginosa* from wound to liver prior to death.

### Experimental Protocol (Mortality Study)

The effect of L. *plantarum* on our burn-sepsis model was tested by giving daily sub-eschar injections of 1x10^9^ cfu (200 μl volume) of *L*. *plantarum* immediately after burn and at Day 2–5, along with the standard *P*. *aeruginosa* injection on Day 2 (24hr after burn). *L*. *plantarum* injection on Day 2 always preceded the *P*. *aeruginosa* injection by 6 hrs. The intensity of Pseudomonal infection was tracked daily using IVIS imaging of bioluminescence. All mice received buprenorphine SQ every eight hours until death/sacrifice (Table 1).

**Table 1:** Experimental outline for Mortality Study, with timing of burn, bacterial injections, and sacrifice.

	Description	#Animals	Day 1	Day 2	Day 3*	Day 4*	Day 5*	Day 6*	Day 7	Day 8*
Control group 1	burn alone	6	burn							sac
Control group 2	burn+Lp	6	burn; Lp	Lp	Lp	Lp	Lp			sac
Exp group 1	burn+Pa	13	burn	Pa						sac
Exp group 2	burn+Pa+Lp	13	burn; Lp	Lp; Pa	Lp	Lp	Lp			sac

* = IVS imaging days

sac = day of sacrifice

Lp = *L plantarum;* dose 1x10^9 cfu

Pa = *P aeruginosa;* dose 1x10^7 cfu.

Four animal cohorts were established: six mice served as control group 1 (burn alone), six mice as control group 2 (burn + daily *L*. *plantarum* only), 13 mice as experimental group 1 (burn + *P*. *aeruginosa* only), and 13 mice as experimental group 2 (burn + *P*. *aeruginosa* + daily *L*. *plantarum*).

The four groups were compared in mortality, quantification of *Pseudomonas* burden in wounds over time (using IVIS imaging of emitted biophotonic signal), quantification of *Pseudomonas* levels in organs (using IVIS imaging), and inflammatory cytokine response levels in the liver (using quantitative reverse transcriptase-PCR [qRT-PCR], see below).

### Experimental Protocol (48hr Challenge)

As initial experiments progressed, it was clear that experimental group 1 animals were dying at different time points (although generally within 48 hours of infection), and control and experimental group 2 animals were allowed to survive to a full week, to be sure no lagging ill effects developed. A uniform and coincident analysis of organs and inflammatory responses across groups was therefore impossible under this scheme. Accordingly, to compare the cohorts at identical time points, a second round of experiments was designed specifically to measure bacterial translocation and inflammatory markers at the 48 hour mark after burn, 24 hours after infection (Table 2).

**Table 2:** Experimental outline for 48 Hour Challenge, with timing of burn, bacterial injections, and sacrifice.

	Description	#Animals	Day 1	Day 2	Day 3*
Control group 1	burn alone	6	burn		sac
Control group 2	burn+Lp	6	burn; Lp	Lp	sac
Exp group 1	burn+Pa	6	burn	Pa	sac
Exp group 2	burn+Pa+Lp	6	burn; Lp	Lp; Pa	sac

* = IVS imaging days

sac = day of sacrifice

Lp = *L plantarum;* dose 1x10^9 cfu

Pa = *P aeruginosa;* dose 1x10^7 cfu.

In this round, six mice served as control group 1 (burn alone), six mice as control group 2 (burn + *L*. *plantarum* only), six mice as experimental group 1 (burn + *P*. *aeruginosa* only), and six mice as experimental group 2 (burn + *P*. *aeruginosa* + *L*. *plantarum*). All animals were burned and received their respective injections and IVIS imaging as described above for the mortality study, except that all animals were sacrificed on the afternoon of Day 3, which corresponds to 48 hrs after burn and 24 hrs after *P*. *aeruginosa* injection. Wounds, liver, and blood were harvested at time of sacrifice. Wounds and liver specimens were subjected to imaging and stored in RNALater for molecular experiments. The liver tissues were subjected to qRT-PCR as below to quantify the mRNA expression of tumor necrosis factor-α (TNF-α), interleukin 6 (IL-6), and interleukin 10 (IL-10). In addition, RT-PCR of a specific *Lactobacillus* rRNA species was used to determine if translocation of *Lactobacillus* to liver could be documented.

### Pseudomonas aeruginosa

*Pseudomonas aeruginosa* Xen41 was obtained from Caliper Life Sciences and was derived from the parental strain *P*. *aeruginosa* PA01. PA cultures were streaked out on Luria broth (LB) agar + tetracycline plates and grown overnight at 37°C. A single colony was selected and grown in 5 mL LB media for 6 hours at 37°C with shaking at 200 rpm until the culture was in log phase growth. Bacteria were diluted based upon previous plating experiments to provide 1x10^7^ cfu in 200 μL of LB media for use in animal experiments. A portion of all diluted bacterial preps was used to enumerate cfus on LB + Tet agar plates to confirm the accuracy of the inoculated dose.

### Lactobacillus plantarum

*L*. *plantarum* was a clinical strain (#10241) obtained from American Type Culture Collection (Manassas, VA). Cultures were streaked out on *Lactobacillus* MRS Broth agar plates (RPI Corp., Mount Prospect, IL) and grown overnight at 37°C. A single colony was selected and grown in 5 mL Lac MRS media overnight at 37°C with 5% CO2 without shaking. Bacteria were diluted based upon previous plating experiments to provide 1x10^9^ cfu in 200 μL of Lac MRS media for use in animal experiments. A portion of all diluted bacterial preps was used to enumerate cfus on Lac MRS agar plates to confirm the accuracy of the inoculated dose.

### IVIS imaging

All living animals underwent IVIS imaging of their wounds on post-injury Days 2, 3, 4, 5 and 7. All animals are anesthetized with inhaled 2% isofluorane for imaging (exposure time of 2 minutes daily). Additionally, the harvested organs (heart, liver, lung, wound) were subjected to IVIS imaging at time of death or at sacrifice on Day 7. The IVIS camera was maintained at standard settings, as follows: imaging mode luminescent, exposure time 1min, binning medium, F/stop 1, Field of View C. Once compiled, images compared quantitatively for relative increase/decrease in bioluminescence using Xenogen Living Image® software.

### Molecular Analysis

#### RNA extraction / purification of samples

Total RNA was purified from a portion of liver tissues stored in RNALater using the RNeasy Mini Kit (Qiagen Inc., Valencia, CA) following manufacturer’s protocols after homogenization using a homogenizer and an on-column DNase treatment step. The concentration of purified RNA was determined using the NanoQuant plate on an Infinite 200 PRO spectrophotometer (TECAN, Switzerland). Quality of RNA extracted was determined by capillary electrophoresis using an Agilent 2100 BioAnalyzer (Santa Clara, CA) and a Nano6000 RNA chip, with all sample RIN values > 6.0.

#### Reverse transcription/ real-time PCR of mouse inflammatory transcripts

The protocol for reverse transcription reactions and real-time PCR was followed as previously described [19,20]. Briefly, 30ng of total RNA purified from mouse liver was used for reverse transcription using MMLV reverse transcriptase (Invitrogen™, Life Technologies, Grand Island, NY), following manufacturer’s protocol and using random primers (Invitrogen™, Life Technologies). Real-time RT-PCR amplification and detection of templates were carried out on an Applied Biosystems PRISM 7900HT system using Applied Biosystems Taqman transcript-specific assays (Applied Biosystems, Foster City, CA) for TNF-alpha (Mm00443260_g1), IL-6 (Mm00446190_m1) and IL-10 (Mm00439614_m1). Using the comparative critical cycle (Ct) method and using GAPDH (Mm99999915_g1) as the endogenous control, the expression levels of the target genes were normalized using a 95% confidence interval.

#### Reverse transcription/ PCR of Lactobacillus-specific RNA from mouse liver

Total RNA was purified from liver from three mice representing each of the four treatment groups. These samples were individually subjected to reverse transcription using the MMLV reverse transcriptase (Invitrogen, Carlsbad, CA) following manufacturer’s protocol utilizing 750 ng RNA and a custom *Lactobacillus*-specific reverse primer (5’- CTTAGATTTGCATAGT -3’). A reverse transcriptase minus reaction was also prepared to ensure that amplification was due to RNA transcription. Custom *Lactobacillus* 16S primers were designed to detect the presence of *L*. *plantarum* using LAC1F (5’- CCGCATAACAACTTGGACCG -3’) and LAC2R (5’- ATACCTGAACAGTTACTCTCAGATA -3’). PCR was carried out using the GoTaq Hot Start Polymerase with green buffer (Promega), 1.5 mM MgCl_2_, 0.5 U Taq, 2.5 μL of RT reaction in a 25 μL PCR reaction and the following cycling parameters: 95°C x 3 min, then 40 cycles of 94°C x 30 sec, 54°C x 30 sec, 72°C x 1 min, with a five minute final 72°C extension. PCR reactions were separated on 2% TAE agarose gels and the resulting primary amplicons of the expected molecular weight of 313 bp were observed through ethidium bromide staining. To confirm assay specificity, 100 ng of purified P. aeruginosa genomic DNA was used as template for PCR and no PCR product was obtained.

### Statistical Analysis

Survival estimates were calculated using the Kaplan-Meier method, with significance calculated from the log-rank approximation of the chi-square test. For the PCR data, repeated measures analysis of variance was used to assess difference in the study groups. A probability (*p*) value of <0.05 was considered significant. No adjustments were performed for multiple comparisons. All analyses were done using Statistical Analysis Software (SAS version 9.3, Cary, NC, USA).

## Results

### A lethal model of burn wound infection

**Fig 1:**
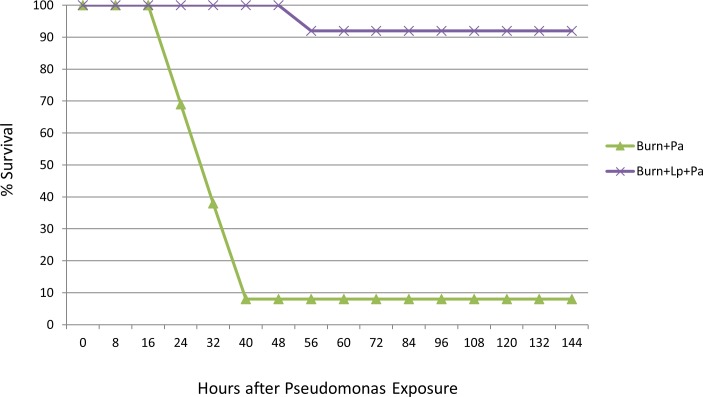
Survival of animals infected with Pseudomonas only versus animals treated with Lactobacillus. Animals receiving the Pseudomonal pathogen alone showed > 90% mortality within 48 hours after Pseudomonal exposure. In contrast, animals receiving probiotic therapy showed < 10% mortality, with a single death in this group occurring at ~56 hours after Pseudomonal exposure, due to apparent sepsis. This difference in mortality was significant (p < 0.0001). All control animals (burn injury alone or burn plus Lactobacillus) showed 100% survival. Lp = *L*. *plantarum*. Pa = *P*. *aeruginosa*.

Our first task was to establish a model where septic translocation following burn injury by a highly relevant clinical pathogen was demonstrably recapitulated, with infection (and not simple burn trauma) as the cause of lethality. This is demonstrated in Fig 1. No mortality (or even morbidity) was observed in either control group (burn injury alone, or burn plus *Lactobacillus*). In contrast, 92% (12/13) of the mice receiving *P*. *aeruginosa* only were dead or moribund within 40 hours of infection; specifically, three animals were noted to have died from apparent overwhelming sepsis, and the remaining nine were sacrificed due to moribund state. These animals demonstrated increasingly sluggish behavior and decreased food intake over this time, culminating in their demise. Weakness of the hindlimb ipsilateral to the infection site was noted within 24 hours, and progressive generalized weakness ensued. The sole surviving mouse in this cohort was permanently paralyzed in the left hindlimb. Thus, infection resulted in 100% mortality or major morbidity.

### Decreased mortality from sepsis with probiotic treatment

Treatment of infected animals with locally applied *Lactobacillus plantarum* (experimental group 2) markedly decreased mortality in this model, from 92% to 7.6% (1/13 animals; p < 0.0001). The 12 surviving mice in this group demonstrated no morbidity whatsoever, such as hindlimb weakness, decreased food intake, etc. The sole animal to succumb in this group did so at a later time than mice receiving *Pseudomonas* alone, suggesting that even here a partially protective effect may have been achieved.

### More rapid clearance of Pseudomonal wound burden with probiotic treatment

**Fig 2:**
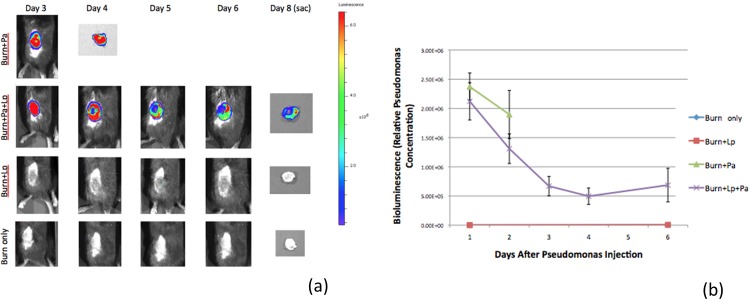
Longitudinal monitoring of Pseudomonal burden in wounds by emitted bioluminescence. (a) Representative images of wounds from animals in the four treatment groups at progressive time points after inoculation. (b) Quantitation of emitted bioluminescence from wounds over time. All animals in experimental groups 1 and 2 demonstrated strong bioluminescence in the wound one day after pathogen exposure. By post-exposure day 2 the amount of emitted light in probiotic-treated animals was clearly declining, and continued to decline over the ensuing several days. Animals receiving pathogen only succumbed to their injuries by post-exposure day 2; the data shown is an amalgam of wound bioluminescence at time of death or sacrifice due to moribund status. No control animals showed any emitted bioluminescent signal. Lp *= L*. *plantarum*. Pa = *P*. *aeruginosa*.

The amount of resident pathogen bioburden in the burn wound itself was measured longitudinally by non-invasive tracking of emitted bioluminescence (Fig 2A and 2B). As expected, no animals in control groups 1 or 2 demonstrated any emitted light from their wounds. All animals in experimental groups 1 and 2 demonstrated strong initial bioluminescence in the wound one day after infection, although even here the probiotic group (experimental group 2) showed slightly less signal. Experimental group 2 (*L*. *plantarum* intervention) also evinced a trend of more rapid decrease in bioluminescence compared to experimental group 1 *(P*. *aeruginosa* only) between 24 and 48 hours post-infection, although this was a somewhat inexact comparison as values for the dying animals in experimental group 1 were collected at the time of their demise, which varied. More importantly, it is clear from the longer time course of experimental group 2 that the Pseudomonal burden in the burn wound continued to decrease over time, with roughly one-quarter of the signal at day 6 after infection as had been present at day 1 after infection.

### Prevention of septicemic accumulation of pathogen in remote organs

**Fig 3:**
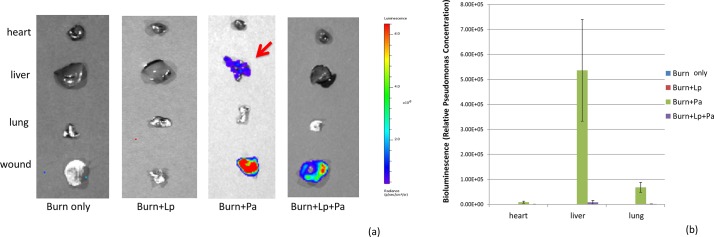
Quantitation of Pseudomonal burden in wounds and remote organs at time of death or sacrifice by emitted bioluminescence. (a) Representative images of harvested tissues from animals in the four treatment groups at time of death or sacrifice. (b) Quantitation of emitted bioluminescence from tissues. No light was seen in any tissues from the control burn only or burn + L. plantarum animals. Strong signal was seen in the wounds and livers of animals in experimental group 1 (burn + Pa). Much reduced signal was seen in animals in experimental group 2 (burn + Lp + Pa). The red arrow in panel (a) highlights clear evidence of bioluminescent pathogen in liver; none such was observed in the surviving animals of experimental group 2, which also showed markedly reduced signal in their wounds. Lung tissue also showed significantly higher levels of pathogen in experimental group 1 compared to experimental group 2. Lp = *L*. *plantarum*. Pa = *P*. *aeruginosa*.

The use of a bioluminescent pathogen allowed us to directly monitor whether *Pseudomonas* from the wound site (as opposed to intestinal or other sources) was translocating and accumulating in remote organs. As expected, no bioluminescence was visualized in the livers, lungs or hearts of the control groups. In experimental group 1 (mice receiving *P*. *aeruginosa* only), 92% (12/13 animals) showed clearly positive bioluminescence in the liver at time of organ harvest and 23% of these demonstrated additional bioluminescence in the lung, showing clearly that a wound-derived septic translocation of pathogen was occurring. In contrast, 92% (12/13 animals) in experimental group 2 (treated with probiotics as well as *Pseudomonas*) demonstrated no detectable bioluminescence in liver or lungs (Fig 3A and 3B). The single mouse in this group that became moribund and died before Day 7 sacrifice did show some bioluminescence in both liver and lung, indicating that in this one instance probiotic treatment ultimately did not prevent sepsis, although it may have delayed it. Quantification of emitted bioluminescence from harvested organs demonstrated statistically significantly higher levels of *P*. *aeruginosa* in the livers, lungs, and even hearts of the animals in experimental group 1 versus experimental group 2 (p < 0.01; Fig 3B).

### Inhibition of the infection-dependent induction of pro-inflammatory cytokines

**Fig 4:**
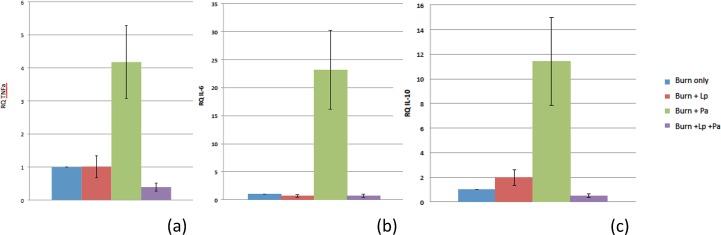
Quantitation of expression of inflammatory mediators in liver by real-time RT-PCR. Quantitation of relative expression of TNF-α mRNA (a), IL-6 mRNA (b), and IL-10 mRNA (c). In all instances, baseline expression of the mediator in question was set at “1” for animals receiving burn injury only. In all instances, Lactobacillus treatment (burn + Lp) showed no or minimal effect on expression levels. In all instances, a marked pathogen-dependent elevation in expression of inflammatory mediators was seen in animals in experimental group 1 (burn + Pa). In all instances, probiotic therapy (burn + Lp + Pa) resulted in reversion to baseline or sub-baseline levels of expression. Lp = *L*. *plantarum*. Pa = *P*. *aeruginosa*.

A characteristic feature of burn sepsis is the systemic hypermetabolic inflammatory response elicited; liver is a target organ involved in this response. In order to determine whether probiotic therapy could suppress such a response, we undertook a second round of experiments wherein all animals were deliberately sacrificed at 48 hrs after burn injury (corresponding to 24 hours after *Pseudomonas* administration in experimental groups 1 and 2). No animals died before this time, and this allowed us to compare the metabolic response in liver at identical times across all four groups (Fig 4A–4C). qRT-PCR assays for TNF-α, IL-6, and IL-10, all inflammatory cytokines previously implicated in burn sepsis, each demonstrated markedly increased expression in the livers of experimental group 1 mice compared to control. This infection-dependent increase was essentially abolished in all cases by probiotic therapy (experimental group 2) (p = 0.006 for TNF-α; p = 0.0003 for IL-6; p = 0.047 for IL-10).

### Distribution of probiotic bacteria to remote organs without apparent harm

**Fig 5:**
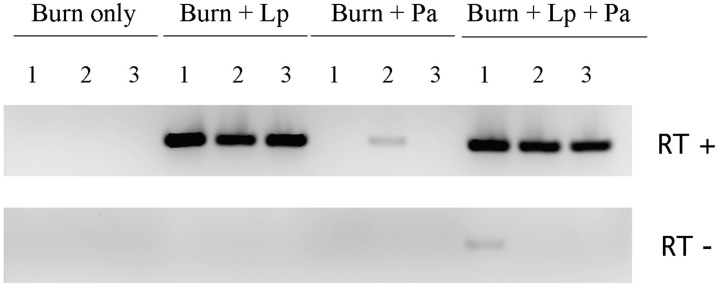
Detection of Lactobacillus RNA in liver. Images of amplicons resulting from RT-PCR for a Lactobacillus-specific 16S rRNA are presented. Results from three animals for each treatment group are shown. Amplicons are of the expected molecular weight (313 bp) and are present in abundance only in animals to whom Lactobacillus was administered. RT minus reactions showed no amplicons in the majority of cases, demonstrating that the amplicons are truly derived from RNA and not contaminating DNA (in one lane, a minor amount of coincident DNA appears to have escaped digestion). Lp = *L*. *plantarum*. Pa = *P*. *aeruginosa*.

In order to determine if our probiotic lactobacilli were translocating from the burn wound, we also assayed the livers of all four groups for expression of an rRNA species unique to *Lactobacillus*. Because bacterial RNA is inherently labile, with a half-life typically measured in minutes, its presence in a tissue is taken as proof of viable bacteria in that tissue. Fig 5 shows that *Lactobacillus* rRNA was abundantly present in liver only when *Lactobacillus* had been administered to the mice, indicating that the inoculated *Lactobacillus* was in fact able to translocate from the wound and lodge in the liver microcirculation, where it failed to induce the pro-inflammatory response engendered by *Pseudomonas* (see above) and where it persisted without any apparent harm to the animal grossly.

## Discussion

Our results clearly indicate that local application of probiotic bacteria can be effective in reducing mortality from burn wound infection and can successfully suppress the systemic hyperinflammatory response such an infection typically provokes. It appears therefore that *Lactobacillus* is somehow able to contravene against the normally invasive Pseudomonal biology. The mechanisms by which this occurs are as yet unclear, but include several possibilities. Probiotic bacteria may physically occupy space in the injured tissues that would otherwise play host to pathogenic bacteria, essentially blocking them from taking residence in the injured tissue. This seems unlikely to be of much import, given that the initial quantum of bioluminescent bacteria measured after infection was only slightly less in probiotic-treated versus pathogen-only animals (see Fig 2B). Another possibility is that the presence of *Lactobacillus* leads to acidification of the surrounding tissue environment, rendering it inhospitable to the Pseudomonal pathogen. This also seems unlikely; the airways of patients with cystic fibrosis have been reported to have relatively acidic pH, but *Pseudomonas* remains the most common pathogen therein encountered. Indeed, in one study, deliberate incubation of *Pseudomonas* at an acidified pH actually decreased its susceptibility to aminoglycoside antibiotic [21]. Moreover, topical treatment of burn wounds in patients with a polylactic acid-acetic acid dressing did not result in any significant decrease in the resident bacterial bioburden and *Pseudomonas* remained one of the most frequently recovered organisms [22].

Another possibility is that the probiotic lactobacilli are actually secreting factors that act to inhibit Pseudomonal physiology. Co-incubation of whole cultures of *L*. *plantarum* with *P*. *aeruginosa* have been reported to decrease the ability of the latter to form biofilm, and also result in decreased production of the acyl-serine homolactone molecules used by *Pseudomonas* for quorum sensing. Similar results are obtained from *L*. *plantarum* culture supernatants, but not from living but washed cells, suggesting that an elaborated factor is the responsible agent. These effects can be obtained even if the supernatant has been pH neutralized, again indicating that simple acidity is not the principal actor [18]. Concentrated supernatant from *L*. *plantarum* has also been shown to inhibit growth and decrease viability of *Escherichia coli*, possibly through disruption of its membrane integrity [23].

Another intriguing possibility is that lactobacilli may directly modulate the host immune system in some way so as to potentiate anti-pathogenic activity and/or inhibit an otherwise damaging inflammatory response. As an example of the former, nasal administration of *Lactobacillus lactis* increased clearance of pathogen from the lung on subsequent infection with *S*. *pneumonia* and resulted in higher serum anti-pneumococcal immunoglobulin production [24]. As to the latter, studies using lactobacilli in models of colitis have shown that there, too, they can suppress inflammatory mediators such as TNF-α and IL-6 [25,26]. Others have demonstrated that lactobacilli can directly suppress secretion of Th1, Th2 and Th17 cytokines by a variety of immune cell types. In one instance, this has been attributed to the ability of lactobacillus to convert L-histidine to histamine, which in turn suppressed TNF-α transcription via activation of the H2 receptor and downstream activation of protein kinase A [27]. Our own studies in a rabbit model of burn wound/infection have shown improved wound healing outcomes with *Lactobacillus* alone compared to control even in the absence of any pathogenic infection, suggesting a direct effect on host biology [Satish et al., manuscript in preparation]. It may be that a variety of elaborated agents from Lactobacilli can be beneficial in modulating the host native and adaptive immune response. Of course, a combination of some or all of the above mechanisms may be in play.

Our results show clearly that topical probiotic therapy can be effective in preventing pathogenic accumulation in distant organs after burn wound infection. Animals inoculated with both *Pseudomonas* and *Lactobacillus* showed no evidence of bioluminescent spread to the liver or lung (excepting in one case) even at six days, despite persistent albeit declining *Pseudomonas* at the wound site. It seems likely that the presence of the probiotic bacteria inhibits septic translocation from the wound, but it is also possible that pathogenic bacteremia is still occurring, but counteracted remotely by some other probiotic effect. Our results also show clearly that *Lactobacillus* itself can and does translocate from the wound, as evidenced by the abundance of *Lactobacillus*-derived RNA in liver tissues, where none would be expected. This RNA only presents in animals where topical probiotic therapy was administered, and is therefore unlikely to be derived from *Lactobacillus* that may have translocated from the gut of the animals. Nonetheless, despite this proof positive of bloodborne dissemination of viable *Lactobacillus* bacteria, none of the mice so treated showed any evidence of systemic shock or ill effect (again excepting the sole animal wherein Pseudomonal sepsis also occurred). It is remarkable that despite abundant viable probiotic bacterial load in the liver, no significant induction of TNF-α, IL-6, or IL-10 is seen. It appears that either the host cells are indifferent to the presence of the probiotic organism, or it may again be that the bacteria themselves are actively suppressing any inflammatory reaction.

These observations in turn suggest that locally applied *Lactobacillus* therapy is likely safe to consider for humans, and that the risk of clinical lactobacillic sepsis is very small. Certainly probiotic bacteria have been widely ingested or administered orally in both home and medical settings, with only a few reported instances of harmful outcome. Many of these derive from a different *Lactobacillus* species (rhamnosus) from that employed here [28], and there is a tendency for the septic episodes to occur in particularly vulnerable patient types, for example children with short gut syndrome [29], bone marrow transplant patients [30], and ulcerative colitis patients being treated with steroids and infliximab [31]. Although burn patients are also thought to be functionally immunocompromised, our results suggest this should be no impediment to probiotic treatment. The possible minor risk of probiotic therapy must also be balanced against the risks of other available therapies. Land et al. describe two patients who became bacteremic with *Lactobacillus* but in whom probiotic therapy was used as a treatment for C. difficile colitis, itself the result of previous heavy antibiotic use for infection/sepsis [32]. It may also be that, in some cases, although *Lactobacillus* was recovered from the patient’s blood, some other unappreciated organism was also present and was actually responsible for any clinical sequelae. While the safety profile of various species of *Lactobacillus* will need to be further explored, our study and the literature would point to a lower relative risk for probiotic compared to antibiotic therapy.

Probiotic therapy has gained increasing enthusiasm, but is usually administered per orem, often for disorders of the gastrointestinal (GI) tract. Burn injury has also been associated with dysregulation of the GI flora, with gut-derived bacteria thought to translocate in response to the burn injury and contribute to subsequent disseminated infections [33]. Probiotics have been shown in animal models to inhibit the systemic spread of gut-derived pathogens and reduce mortality, for example in the setting of cyclophosphamide use [34]. Furthermore, one study examined 56 burn patients, half of whom received oral probiotics and half of whom did not. The investigators noted significantly fewer deaths in the treated group among patients with large (41–70%) total body surface area (TBSA) burns, and suggested that probiotic food additives may be clinically beneficial in these patients [35] It is unclear whether local probiotic therapy to the wound as employed here would confer a similar benefit versus gut-derived pathogens, but given that the probiotic bacteria themselves appear to translocate it is possible that it could be used in combination with oral therapy.

The management of burn injuries to limit infection and resurface the wound currently relies on several core strategies: deeper (full-thickness) injuries usually require surgical excision with skin replacement through autografts, allografts, temporary dressings or skin substitutes. Systemic antibiotics are sometimes adjunctively used, although some practitioners refrain until evidence of invasion or sepsis for fear of selecting for resistant microorganisms, a growing problem in burn units nationwide. Moreover, prolonged use of such antibiotics may predispose to fungal superinfection, which can be devastating. Topical antimicrobials, especially silver-containing compounds such as silver sulfadiazine, have been the mainstay of local burn wound therapy for over 40 years [36]. Progress over this time has been limited and incremental, with most effort devoted to deploying novel carriers for the silver, or extending its use to other types of wounds. However, recent reports have noted multiple drawbacks to the use of topical silver as an antimicrobial: development of resistance (including multi-drug resistance) by induction of an efflux mechanism (including in *P*. *aeruginosa*), local and systemic toxicity to host tissues (eg. renal toxicity, hepatotoxicity), argyria, and dyschromia of the skin [37–39]. In addition, some reports have questioned the clinical efficacy and cost-efficacy of topical silver [40]. Furthermore, although silver is frequently touted as an effective anti-biofilm agent, it is also clear that biofilms can persist even in the presence of silver [41,42]. In this context, there is a pressing need for the development of new therapeutic modalities which can be administered to the infected burn wound that will not elicit harmful side effects and that will facilitate wound healing.

Locally applied probiotic bacteriotherapy offers an attractive if counterintuitive means to address the problem of burn wound infection. Probiotic bacteria may be active against a range of pathogens simultaneously, including drug-resistant organisms. They have demonstrated activity against fungal pathogens as well, for example, in their use as a treatment of Candidal vulvovaginitis [43]. They are unlikely to facilitate emerging antibiotic resistance and would be potentially effective even against pathogens in biofilm configuration. They have the ability to independently exert beneficial effects on host cells and tissues and to suppress the damaging hyperinflammatory response burn injuries frequently elicit. They are inexpensive and could be easily applied topically to a burn injury site. We anticipate that in actual clinical use, local probiotic bacteriotherapy could be best deployed immediately after surgical debridement of the wound has reduced the pathogenic bioburden to a much lesser level, although there is nothing to preclude use immediately after injury as well. Because *Lactobacillus* has a remarkable property of retaining viability after dehydration/rehydration, it may be possible to arrive at an off-the-shelf probiotic therapy; indeed, alginate film carrying *Lactobacillus* active against *Pseudomonas* has already been described [44].

Thus far, the use of topical probiotics in burn patients is limited to a single report. Peral et al. applied topical *L*. *plantarum* soaked into gauze sponges to a cohort of second and third degree burn injured patients, divided into early and late treatment groups, and compared outcomes to a similar cohort of patients treated with silver sulfadiazine cream [45]. No systemic antibiotics were used, and the majority of patients had TBSA burns of <15%. The numbers of patients were too small to achieve statistical significance, but the investigators found little to no difference in the two cohorts overall in the rate of healing or in the bacterial counts in the healing wounds on subsequent biopsy. Importantly, no adverse outcome occurred in the 38 patients (with varying depth and time of injury) that could be related to the use of *Lactobacillus*, including no difference in subsequent skin graft take. These extremely preliminary data give promise that an appropriate regimen of local probiotic bacteriotherapy for burn injured patients can be safe and at least as effective as silver sulfadiazine. Our own results highlight that local probiotic therapy can dramatically mitigate the risk of sepsis and death, and thus may be even more beneficial for the patients with larger TBSA burns that are most at risk for these complications. Further work to elucidate the mechanisms by which probiotic bacteria exert their effects on pathogens and hosts, as well as further experience with clinical use of local bacteriotherapy in burn (and other) wound scenarios, will clarify the expanding role of probiotic therapy.
